# Association Between Antihypertensive Medications and Fracture Risk in Elderly Patients: A Cross-Sectional Study

**DOI:** 10.7759/cureus.69714

**Published:** 2024-09-19

**Authors:** Muhammad D Nadeem, Junaid Ali, Shahin Shah, Abroo Mahmood, Umair Ahmad

**Affiliations:** 1 Medicine, University Hospitals Birmingham NHS Foundation Trust, Birmingham, GBR; 2 General Medicine, Khyber Medical University, Peshawar, PAK; 3 General Medicine, Medlife Medical Center, Abu Dhabi, ARE; 4 Primary Care, Advocare Northbrunswick Medical Associates, North Brunswick, USA; 5 Medicine, Khyber Pakhtunkhwa Health Department, Peshawar, PAK

**Keywords:** antihypertensive medications, diuretics, elderly, falls, fracture risk, hypertension, osteoporosis

## Abstract

Background

The use of antihypertensive medications is common among older adults to manage hypertension and prevent cardiovascular events. However, the potential impact of these medications on bone health and the risk of fractures remains a concern. This study investigates the association between antihypertensive medication use and fracture risk in elderly individuals.

Materials and methods

A cross-sectional study was conducted from February 2023 to July 2024, including 299 elderly patients (aged ≥65) diagnosed with hypertension and currently using antihypertensive medications. Data were collected from medical records, focusing on demographics, fracture incidence, comorbid conditions, and medication use. Logistic regression models were used to analyze the association between antihypertensive use and fracture risk, adjusting for confounders.

Results

Among the participants, 110 reported falls, and 88 (29.43%) sustained fractures. Fractures were more prevalent among females (63.6%) and those aged 75-84 years (45.5%). A history of falls, mobility difficulties, osteoporosis, and urinary incontinence were significantly associated with fractures. While most antihypertensive classes did not show a significant association with fracture risk, diuretics were associated with a 2.3-fold increased risk of fractures (OR 2.30, p=0.037).

Conclusions

This study highlights the need for careful consideration of fracture risk in elderly patients using antihypertensive medications, particularly diuretics. Healthcare providers should balance the benefits of blood pressure control with the potential risk of fractures in this population.

## Introduction

Hypertension is prevalent among older adults, and guidelines for reducing the risk of myocardial infarction (MI) and stroke include the regulation of blood pressure [1]. The use of antihypertensive medications is common among older adults to manage hypertension and prevent cardiovascular events. Emerging evidence indicates that these medications may impact bone health, thereby influencing the risk of fractures in the elderly population [2].

When determining which antihypertensive drugs to start, maintain, or modify in older individuals with various underlying conditions, it is essential to carefully consider the possible advantages in comparison to the associated dangers [3]. Antihypertensive medication in clinical trials has shown a 28% decrease in the relative risk of cardiovascular events. This treatment lowers the absolute risk of cardiovascular disease in participants over a period of 4.5 years. Nevertheless, the individuals included in these studies often had a lower number of additional health disorders compared to the overall aged population [4]. This raises uncertainty over whether the reported advantages apply to older persons with numerous chronic illnesses [5].

The ideal levels of blood pressure management in older persons are still unclear since studies demonstrate inconsistent associations between the extent of blood pressure decrease and cardiovascular advantages [6]. In addition, individuals with numerous medical issues may be more susceptible to experiencing negative side effects from antihypertensive drugs as compared to the relatively healthy participants in randomized controlled trials. The possibility of these drugs increasing the risk of severe fall injuries, such as traumatic brain damage and hip fractures, is concerning, as these injuries can significantly affect functionality and mortality, though not always to the same extent as cardiovascular events [7].

There is evidence indicating that antihypertensive drugs may increase the likelihood of falls and associated injuries. Common side effects such as difficulties with balance and walking, feelings of dizziness, and low blood pressure while changing positions are important variables that increase the chance of falling and experiencing fractures [8]. A meta-analysis of observational studies demonstrated that the use of antihypertensive medicines was linked with a 24% higher likelihood of falling [9]. However, it is important to note that the included studies differed in how they accounted for variables that may influence the results and in how they identified fall-related outcomes. Moreover, studies investigating the correlation between the commencement of various antihypertensive drugs and the incidence of falls and fractures have shown inconclusive findings. The relationship between continuous usage of antihypertensive medication and the likelihood of experiencing severe fall injuries is still not well understood [10].

Given the aging population and the widespread use of antihypertensive drugs, understanding their potential role in increasing fracture risk is critical for improving patient safety. The purpose of this article is to examine the existing data about the link between the use of antihypertensive medicine and the risk of fractures in older individuals. The goal is to offer healthcare providers valuable information to help them strike a balance between managing hypertension and reducing the likelihood of fractures.

## Materials and methods

This study utilized a cross-sectional design to assess the risk of fractures among elderly individuals diagnosed with hypertension. The data collection spanned from February 2023 to July 2024, covering a comprehensive period to ensure the inclusion of a diverse sample of patients. This design allowed for a snapshot of the relationship between antihypertensive medication use and fracture risk within a defined timeframe.

The study focused on elderly patients aged 65 and older, who were diagnosed with hypertension and were either admitted to the hospital wards or visited the emergency department during the study period. The study population was drawn from both inpatient and outpatient settings, ensuring a broad representation of individuals with varying levels of hospital interaction. 

The inclusion criteria for this study required participants to be elderly adults aged 65 and above who had been diagnosed with hypertension and were currently taking antihypertensive medications. Participants were eligible if they were admitted to hospital wards or visited the emergency department for any reason during the study period. The exclusion criteria ruled out any individuals who had incomplete medical records, were not on antihypertensive medication, or had secondary causes of hypertension. Additionally, patients with severe cognitive impairment that could interfere with the study's data collection process were excluded. These criteria ensured that the study focused on a specific population of elderly hypertensive patients to investigate the associated risk factors accurately. The convenience sampling technique was utilized to collect the study sample.

Data was meticulously collected from patients' medical records using a data collection form designed based on existing literature to ensure comprehensive and accurate information capture. The information extracted included several key areas. Patient demographics were recorded, covering basic characteristics such as age, gender, and socioeconomic status. Detailed fracture information was also gathered, including the fracture types, locations, and the circumstances surrounding their occurrence. Comorbid conditions that could impact fracture risk were documented to provide a full picture of each patient's health status. Medication use was carefully noted, with specific attention to antihypertensive medications and any other relevant drugs. Reports of falls by the patients were included to assess their frequency and potential impact on fracture risk. Clinical parameters were also collected, such as blood pressure levels and other vital statistics pertinent to hypertension management. To validate the accuracy of extracted data, two independent reviewers conducted the extraction, resolving discrepancies through consensus. Fall reports were cross-checked with clinical records, while comorbidities were verified using diagnostic codes. Medication use was validated by reviewing prescription records and pharmacy logs to ensure accurate data on antihypertensive usage and adherence. The Institutional Review Board (IRB) of the Public Health Institute, Peshawar (Re: IPH&SS-AAA-325) approved the current study research protocol. The participants were given verbal consent before data collection.

Descriptive statistics were used to summarize the demographic and clinical characteristics of the study population. The association between antihypertensive medication use and fracture risk was analyzed using logistic regression models, adjusting for potential confounders such as age, gender, comorbidities, and medication use. Results were reported as odds ratios (ORs) with corresponding 95% confidence intervals (CIs). p< 0.05 was considered statistically significant.

## Results

In the study sample, males accounted for 156 (52.1%) of the participants, while females made up 143 (47.9%). Participants aged 6-74 years comprised 148 (49.5%) of the total, with 92 (30.7%) aged 75-84 years, and 59 (19.8%) over 85 years old. Regarding BMI, 120 (40.1%) had a normal weight, 92 (30.7%) were overweight, and 61 (20.4%) were classified as obese, while 26 (8.7%) were underweight. A significant proportion of participants, 178 (59.5%), reported a history of falls, compared to 121 (40.5%) who did not report falls (Table 1).

**Table 1:** Demographic and clinical characteristics (n=299) BMI: Body Mass Index

Variables	N	%
Gender		
Male	156	52.1%
Female	143	47.9%
Age		
60-74 years	148	49.5%
75-84 years	92	30.7%
>85 years	59	19.8%
BMI		
Underweight	26	8.7%
Normal	120	40.1%
Overweight	92	30.7%
Obese	61	20.4%
History of fall		
Yes	178	59.5%
No	121	40.5%

**Figure 1:**
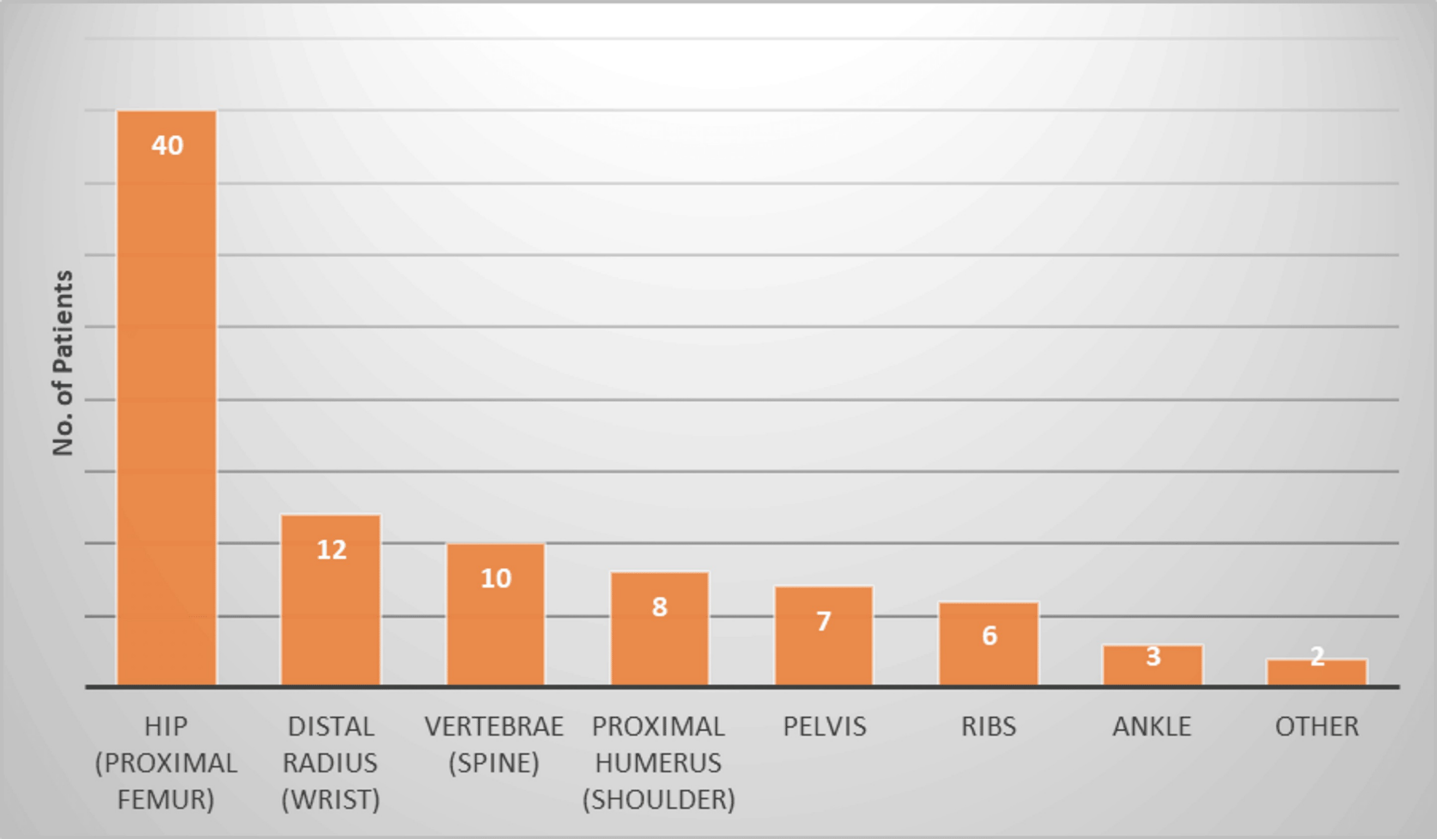
Distribution of fracture locations (n=88)

Among 299 elderly patients, 110 reported experiencing falls, and 88 (29.43%) of these individuals sustained fractures. The fractures were distributed as follows: 45.5% had hip fractures (proximal femur), 13.6% had distal radius (wrist) fractures, 11.4% had vertebral (spine) fractures, and 9.1% had proximal humeral (shoulder) fractures (Figure 1).

This study analyzed several factors to determine their association with fractures in elderly patients, as depicted in Table 2. The age distribution showed a significant difference, with patients aged 75-84 years having the highest proportion of fractures (45.5%), followed by those aged 65-74 years (34.1%) and over 85 years (20.4%), indicating a higher fracture risk with increasing age (p=0.041). Gender also played a role, as fractures were more common in females (63.6%) than males (36.4%), with a statistically significant difference (p=0.03). Although BMI showed no significant difference between the groups (p=0.060), underweight individuals had a higher proportion of fractures (13.6%) compared to those without fractures (6.6%). A history of falls was nearly significant (p=0.051), with a higher percentage of fall history among those with fractures (68.2%). Poor perceived health status, mobility difficulties, urinary incontinence, cognitive impairment, and osteoporosis were significantly more prevalent among patients with fractures, highlighting their strong association with fracture risk (p-values ranging from 0.001 to 0.014). In contrast, other variables, such as prior MI, diabetes mellitus, and heart failure, did not show significant differences between the groups. The use of antihypertensive medications, including different classes, also did not significantly differ between those with and without fractures.

**Table 2:** Demographic and health-related factors among study samples reported with and without fractures BMI: Body Mass Index; CVI: Cardiovascular Injury; MI: Myocardial Infarction; ARBs: Angiotensin Receptor Blockers; ACE: Angiotensin Co-enzyme; *: p< 0.05, **: p< 0.01

Variables	With Fractures (n=88)	Without Fractures (n=211)	p-value
Age			0.041*
65-74 years	30 (34.1%)	90 (42.7%)	
75-84 years	40 (45.5%)	80 (37.9%)	
>85 years	18 (20.4%)	41 (19.4%)	
Gender			0.03*
Male	32 (36.4%)	78 (36.9%)	
Female	56 (63.6%)	133 (63.1%)	
BMI			0.060
Underweight (<18.5)	12 (13.6%)	14 (6.6%)	
Normal (18.5-24.9)	30 (34.1%)	90 (42.7%)	
Overweight (25-29.9)	28 (31.8%)	64 (30.3%)	
Obese (≥30)	18 (20.5%)	43 (20.4%)	
History of fall			0.051
Yes	60 (68.2%)	118 (55.9%)	
No	28 (31.8%)	93 (44.1%)	
Perceived health status			0.002**
Excellent/Good	35 (39.8%)	124 (58.8%)	
Fair/Poor	53 (60.2%)	87 (41.2%)	
Mobility difficulty	58 (65.9%)	92 (43.6%)	0.001**
Urinary incontinence	50 (56.8%)	84 (39.8%)	0.005**
Cognitive impairment	42 (47.7%)	68 (32.2%)	0.014*
Uses assistive device	44 (50.0%)	62 (29.4%)	0.001**
Osteoporosis	55 (62.5%)	84 (39.8%)	0.001**
Prior MI	20 (22.7%)	40 (18.9%)	0.441
Diabetes mellitus (DM)	30 (34.1%)	60 (28.4%)	0.316
Heart failure	25 (28.4%)	40 (18.9%)	0.071
Other CVI history	18 (20.4%)	38 (18.0%)	0.630
Medications other than antihypertensives	40 (45.5%)	80 (37.9%)	0.213
Class of antihypertensive used			
ACE inhibitors	20 (22.7%)	55 (26.1%)	0.561
ARBs	18 (20.4%)	45 (21.3%)	0.852
Beta-blockers	16 (18.2%)	39 (18.5%)	0.944
Calcium channel blockers	15 (17.0%)	32 (15.2%)	0.688
Diuretics	12 (13.6%)	28 (13.3%)	0.027*

The results of logistic regression models, adjusting for potential confounders such as age, gender, comorbidities, and other factors, are shown in Table 3. Most classes, including ACE inhibitors, angiotensin receptor blockers (ARBs), beta-blockers, calcium channel blockers, and alpha-blockers, did not show statistically significant associations, as indicated by their p-values greater than 0.05 and confidence intervals of 1. This suggests that these medications neither significantly increase nor decrease the risk of fractures. However, diuretics were associated with a statistically significant increase in risk, as evidenced by an OR of 2.30 and a p-value of 0.037.

**Table 3:** Regression analysis ACE: ACE: Angiotensin Co-enzyme; ARBs: Angiotensin Receptor Blockers; *: p< 0.05

Antihypertensive Class	Odds Ratio (OR)	95% Confidence Interval (CI)	p-value
ACE inhibitors	1.15	0.69-1.93	0.561
ARBs	1.09	0.64-1.85	0.852
Beta-blockers	1.03	0.49-2.11	0.944
Calcium channel blockers	1.12	0.60-2.08	0.688
Diuretics	2.30	0.40-2.37	0.037*
Alpha-blockers	1.35	0.32-5.61	0.707
Other antihypertensives	0.98	0.55-1.76	0.339

## Discussion

The present study aimed to assess the prevalence of fractures among elderly patients undergoing treatment with antihypertensive medications, revealing that 29.43% of the study sample had sustained fractures due to falls. These results are consistent with existing literature, which emphasizes a significant association between antihypertensive medication use and an elevated risk of fractures among older adults. Previous research has reported similar trends, particularly noting an increase in hip fractures shortly after the initiation of antihypertensive therapy, especially within the first 45 days of treatment [11,12]. This heightened risk is likely due to potential side effects of these medications, such as orthostatic hypotension, which can cause dizziness and lead to falls.

The gender distribution in this study, where males constituted 52.1% and females 47.9%, reflects typical demographic trends in elderly populations. However, the higher prevalence of fractures among females (63.6%) compared to males (36.4%) aligns with other studies that highlight the increased fracture risk in postmenopausal women, primarily due to lower bone density and hormonal changes [13,14]. Age was also a significant factor, with individuals aged 75-84 years showing the highest proportion of fractures (45.5%), supporting the well-documented association between advanced age and fracture risk due to decreased bone density and increased fall susceptibility in older adults [15].

Regarding BMI, while no statistically significant difference in fracture risk was found between groups, the study did observe a higher incidence of fractures among underweight individuals, consistent with research linking low BMI to an increased risk of osteoporosis and fractures [16,17]. Interestingly, the lack of a significant association between obesity and fractures contrasts with some studies that suggest higher BMI may increase fracture risk due to greater mechanical loading on bones [18].

The study found that a history of falls was nearly significant in predicting fractures, with 68.2% of those with fractures reporting prior falls. This finding is consistent with extensive research identifying fall history as a critical predictor of future fractures in elderly populations. However, the slight difference in significance may be due to sample size or other confounding factors [8,19,20]. Additionally, the study identified significant associations between fractures and various health-related factors, including poor perceived health status, mobility difficulties, urinary incontinence, cognitive impairment, and osteoporosis. These findings align with existing literature emphasizing these factors as key contributors to fracture risk. For example, a study by Hellman-Bronstein et al. underscores the role of mobility difficulties and cognitive impairment in increasing fracture susceptibility, suggesting these conditions can lead to falls and subsequent fractures [21]. Similarly, Yoryuenyong et al. found that urinary incontinence is associated with a higher incidence of fractures, likely due to its impact on balance and mobility [22].

Moreover, Sultan and Bukhari, in their study, have highlighted the direct correlation between osteoporosis and fracture risk, reinforcing the importance of bone health in preventing fractures [23]. Furthermore, Valentin et al. demonstrated that poor perceived health status is a significant predictor of fracture risk, suggesting that individuals with a negative health perception may engage in less preventive behavior [24]. Collectively, these studies affirm the multifaceted nature of fracture risk, underscoring the need for comprehensive assessment and intervention strategies.

Interestingly, while most antihypertensive medications did not show a significant association with fracture risk, diuretics were linked to a higher risk. This finding supports the growing body of evidence indicating that while some antihypertensive medications may be neutral regarding fracture risk, diuretics, particularly those causing electrolyte imbalances, may increase fracture susceptibility [25]. Additionally, some research suggests that the risk of fractures may vary depending on the type of antihypertensive medication used. For instance, studies have indicated that calcium channel blockers like verapamil and diltiazem might reduce fracture risk in elderly hypertensive patients, highlighting the complex relationship between antihypertensive medications and fracture risk [26]. This study has a few limitations that should be acknowledged. Self-reported data on falls and health status may introduce bias, and the lack of bone density measurements restricts the assessment of osteoporosis-related fracture risk. Additionally, the study did not account for medication adherence or variations in antihypertensive drug types and dosages, which could influence the results. In addition, polymedication, which could influence both fall risk and fracture outcomes, was not fully accounted for.

## Conclusions

Serious injuries like hip fractures and head injuries carry morbidity and mortality rates similar to cardiovascular events. While this observational study cannot establish a cause-and-effect relationship and potential confounding factors cannot be ruled out, the findings indicate an association between antihypertensive medications and an elevated risk of serious fall injuries and fractures, as evidenced by 29.4% of our study sample sustaining major fractures. Therefore, the risks and benefits of continuing antihypertensive medications in older adults with chronic conditions should be carefully considered.
